# Letter from the Editor-in-Chief: Irreproducible Results

**DOI:** 10.4303/jdar/235879

**Published:** 2014

**Authors:** Michael J. Kuhar

**Affiliations:** Yerkes National Primate Research Center, Emory University, 954 Gatewood Rd NE, Atlanta, GA 30329, USA

Dear Colleagues,

As you probably know, significant concern has developed over the irreproducibility of published results, and the NIH has expressed serious concern over this problem [1, 4].

There is no real worry that this is due to scientific misconduct. Rather, it is believed to be due to poor training of investigators in experimental design, lack of reporting of methodologies in published papers, lack of critical evaluation and reviewing, and lack of publications on negative data or on critiquing others’ methods. Other forces such as various kinds of bias presumably contribute to this as well. There are a number of papers on this topic and I cite a few that may be helpful [1, 2, 3, 4, 5].

Preclinical studies are more of a concern than clinical studies, where standardized reporting procedures exist and where there is more rigorous design and oversight [1]. The use of animal models in preclinical work seems to be an area where problems seem to crop up more often. The use of different strains of animals, for example, can be a source of disagreement. Preclinical work may be an area where rapid progress can be made.

Obviously, something must be done, and it is being done. The NIH is developing a number of initiatives, some of which will become mandatory in NIH sponsored training programs. There will be specific training opportunities with an emphasis on good experimental design. A checklist will be developed for reviewers and evaluators that address experimental procedures such as sample size calculations, randomization, blinding and so forth. Access to raw data and increased transparency will be addressed. Other groups in the scientific community will have to participate as well. These groups will include journals, private granting agencies, various review panels, and others.

Journal of Drug and Alcohol Research (JDAR) has not had a problem with irreproducible results, but it is not a problem that can be ignored. I write this letter because this is a significant issue that is not going away. Correcting this problem can only be a good thing. This journal will support efforts to improve reproducibility, and will judiciously follow recommendations made by responsible groups. It seems reasonable to suggest that readers and submitters follow this topic and the recommendations from the NIH and elsewhere. Everyone—authors, editorial staff, readers, and reviewers—want JDAR to be a solid and trustworthy journal.
